# Dr. John Bellinger

**Published:** 1860-11

**Authors:** 


### NECROLOGICAL NOTICES.

DR. JOHN BELLINGER died on the thirteenth of August, of pulmonary consumption, at Charleston, South Carolina, in the fifty-sixth year of his age. He received his diploma at the University of Pennsylvania, in 1826, and immediately moved to Charleston, where, after many privations, he finally succeeded in gaining an enviable reputation as a practitioner of medicine and surgery. In 1848 he was appointed to the chair of surgery in the Medical College of the State of South Carolina, and filled the post to the entire satisfaction of his colleagues and his classes until 1851, when he voluntarily resigned.

